# Caveolin-1 as a target in prevention and treatment of hypertrophic scarring

**DOI:** 10.1038/s41536-019-0071-x

**Published:** 2019-04-26

**Authors:** Ilja L. Kruglikov, Philipp E. Scherer

**Affiliations:** 1 Scientific Department, Wellcomet GmbH, Karlsruhe, D-76129 Germany; 2 Touchstone Diabetes Center, University of Texas Southwestern Medical Center, Dallas, TX 75390-8549 USA

**Keywords:** Diabetes complications, Type 2 diabetes

## Abstract

Reduced expression of caveolin-1 (Cav-1) is an important pathogenic factor in hypertrophic scarring (HTS). Such a reduction can be found in connection with the main known risk factors for HTS, including dark skin, female gender, young age, burn site and severity of the injury. The degree of overexpression of Cav-1 associated with different therapeutic options for HTS correlates with clinical improvements in HTS. This makes endo- or exogenous induction of Cav-1 not only an important therapeutic target for HTS, but also highlights its use as a preventive target to reduce or avoid HTS formation.

## Introduction

Fibrosis is a tissue response to injury generally preceded by a long-term inflammatory reaction and connected with excessive deposition of extracellular matrix, especially of collagen. In the skin, fibrosis can lead to production of hypertrophic scars (HTS) which are typical for the full thickness skin injury^1^ and appear in 30–72% of patients following thermal injury.^2^ The main risk factors in HTS include dark skin, female gender, and young age.

Different types of non-surgical treatments have been applied to the treatment of HTS, among them various kinds of static and dynamic mechanical forces, light-based therapies, and application of different injectable and topical drugs, including corticosteroids, chemotherapeutics, and immune-modulators.^3,4^ Recently it was reported that intralesional injections of hyaluronidase can also effectively reduce HTS.^5^ None of these treatments was confirmed to be effective in the prevention of initial HTS formation.

It is widely accepted that transforming growth factor beta (TGF-β) is a master regulator of fibroblast activation and fibrosis.^6^ However, it is not clear how the TGF-β pathway is connected with the main risk factors. Neither is it known how it affects the clinical effectiveness of the various non-invasive treatment strategies applied for the reduction of HTS.

TGF-β signaling is tightly connected with plasma membrane structures known as caveolae. Caveolae are characteristic Ω-shaped plasma membrane invaginations forming the nanodomains with typical sizes of 50–100 nm which are present in different cells but especially highly abundant in mechanically stressed cells, such as endothelial cells, fibroblasts, adipocytes, and muscle cells, where they constitute up to 50% of the total surface area and can exist as single invaginations or clusters.^7^ These nanodomains play an important role in rapid regulation of cellular volumes, cell adhesion, different signal transduction processes, as well as in the processes of endo- and exocytosis.^8^ Depending on the cell type, caveolae contain different types of caveolins (Cav-1 to 3), all of which are known to be involved in the processes of local proliferation and inflammation in various tissues. Importantly, Cav-1 is involved in the regulation of TGF-β signaling by means of a physical interaction with TGF-β membrane receptors^9^ and participates in the internalization of these receptors.^10^ Internalized TGF-β receptors undergo rapid degradation, thereby leading to an effective reduction of TGF-β signaling. Such interactions explain the negative correlation between Cav-1 expression and TGF-β activity observed in pulmonary and dermal fibrosis.^11,12^ Accordingly, induction of Cav-1 expression leads to suppression of TGF-β signaling and an improvement of fibrosis.^10^

Recently, the regression of HTS was connected with an induction of endothelial dysfunction causing atrophy of endothelial cells.^13^ This is consistent with previous reports that the administration of angiogenesis inhibitors can reduce HTS formation. At the same time, angiogenesis inhibitors can form a complex with Cav-1,^14^ and Cav-1 inhibits endothelial cell proliferation by inducing a cell cycle arrest in the G0/G1 phase.^15^ These results additionally support the interrelation between Cav-1 expression and HTS formation and regression, and demonstrate that low local Cav-1 expression may be an important pathophysiological factor in HTS.

Here, we connect the risk factors in HTS and structural modification of these scars observed after application of different physical and pharmacological agents with increased expression of Cav-1 in the skin and subcutaneous white adipose tissue (WAT) underneath the lesion-containing skin and discuss whether Cav-1 has the potential to be a therapeutic target in HTS treatment. For this, we will first consider the possible involvement of Cav-1 in some important epipathogenic factors in HTS formation.

## Caveolin in inflammation and collagen synthesis

Local tissue inflammation and overexpression of collagens are the typical hallmarks of HTS. Caveolins are significantly involved in both processes.

### Cav-1 in inflammation

There are a number of different observations that implicate Cav-1 and Cav-2 in the regulation of local tissue inflammation.^16^ Cav-1^−/−^ mice are characterized by a low-grade systemic proinflammatory status.^17^ The elimination of Cav-1 promotes the polarization of M2 macrophages in mice.^18^ Whereas macrophages of the M1 subtype secrete mediators promoting inflammation, M2 macrophages are known to suppress inflammation and promote fibrosis. Both of these processes must be substantially involved in HTS formation, since systemic macrophage depletion in the subacute phase of wound healing caused by intraperitoneal injections of clodronate significantly reduced HTS formation.^19^ The dermis of Cav-1^−/−^ mice is indeed strongly infiltrated with macrophages and autophagic cells,^20^ and it was reported that the ratio of M1 vs. M2 macrophages in the dermis of keloids is significantly shifted to the M2 subtype.^21^ Moreover, Cav-1^−/−^ mice display an almost complete absence of the dermal WAT (dWAT) layer^22^ which is involved in inflammatory skin reactions.^23–25^ Additionally, Cav-1 deficiency provided reduced trafficking of dendritic cells to lymph nodes.^26^ Glucocorticoid receptors are co-localized with caveolae and downregulation or ablation of Cav-1 lead to impaired functioning of glucocorticoids.^27^

These observations underline that Cav-1 is not only a marker, but also the target for inflammation.

### Cav-1 in collagen expression

We appreciate that the local expression of Cav-1 and collagen 1 (Col1) are negatively correlated. This correlation is especially pronounced in scleroderma,^20^ keloids,^28^ and HTS.^29^ Moreover, Cav-1^−/−^ mice demonstrate significant shift in the synthesis/degradation balance of collagens towards an increase in net synthesis, which correlates with increased local density of myofibroblasts within the skin and the enhanced cell death and fibrosis observed in WAT.^30^

Myofibroblasts, which are strongly involved in excessive collagen production in HTS, have a high-level expression of the contractile marker α-SMA. These cells typically demonstrate significantly lower levels of Cav-1 expression compared to fibroblasts, which led to the suggestion that there is a negative correlation between Cav-1 and TGF-β expression.^11,12^ Upon comparing normal fibroblasts and fibroblasts from fibrotic lesions in scleroderma subjects (myofibroblast-rich population of cells), these cells react very differently to the modulation of Cav-1 expression.^31^ A reduction of Cav-1 expression induces increased α-SMA expression only in normal fibroblasts expressing relatively low levels of α-SMA, but not in myofibroblasts which already express the high levels of this marker. On the other hand, an increase in Cav-1 expression decreases the α-SMA expression in scleroderma fibroblasts, but not in normal fibroblasts.^31^

These results support the idea that Cav-1 is not merely involved in the regulation of collagen production by myofibroblasts, but may also play a part in the differentiation process of these cells and thus may be directly involved in the pathogenesis of HTS.

### Cav-1 in the regulation of heat shock proteins

Fibro-proliferative diseases are characterized by overexpression of some heat shock proteins (HSPs) which are involved in the inflammatory response of the tissue as well as in collagen synthesis.^32,33^ For example, Hsp27 (up to 10-fold), Hsp47 (up to 16-fold) and Hsp70 (up to 3-fold) were found to be strongly expressed in keloid tissue.^32^ Hsp27 is a cellular differentiation marker, which can affect the formation of actin microfilaments and regulate endothelial cell migration. Hsp47 is a collagen-specific molecular chaperone, playing a critical role in the biosynthesis and secretion of procollagens. Its overexpression in keloid fibroblasts can induce excessive collagen accumulation through increased collagen synthesis, as demonstrated both in vitro^34^ and in vivo.^35^ Hsp70 is a multifunctional chaperone responsible for regulating refolding of misfolded proteins and degradation of unstable proteins, and the various Hsp70 isoforms are distributed in different intracellular, plasma membrane and extracellular compartments. A potent suppression of HSP-production in some diseases (e.g., diabetes) highly correlates with delayed wound healing^36^ and HSPs may, therefore, be targets to enhance this process.^37^ At least some HSPs were shown to be co-localized with caveolae in the plasma membrane of different cells,^38,39^ and HSP activity correlates with expression of Cav-1.^39^

Recent observations suggest that Hsp27 is critically involved in bleomycin-induced pulmonary fibrosis, thereby influencing the differentiation of lung fibroblasts into myofibroblasts and the overproduction of Col1.^40^ An induced siRNA knockdown of Hsp27 provided by these authors demonstrated effective suppression of bleomycin-induced pulmonary fibrosis. At the same time, Cav-1 was reported to be a negative regulator of ERK1/2-Hsp27 signaling, thereby influencing the uptake of exosomes and modifying the exosomal exchange between the cells.^41^

Strong negative correlations between Cav-1 and Hsp47 were very recently reported in myocardial fibrosis.^42^ Injections of a peptide containing the Cav-1 scaffolding domain led to reversing of Cav-1 deficiency in the tissue and to a significant reduction of expression levels of Col1 and the collagen chaperone Hsp47. Moreover, the anti-fibrotic and anti-inflammatory effects of pirfenidone (used in the treatment of idiopathic pulmonary fibrosis) were not only connected with an inhibition of Col1 expression, but also with a suppression of Hsp47 expression in lung fibroblasts.^43^ In fact, pirfenidone significantly increases the protein expression levels of Cav-1 in lung tissue subjected to bleomycin-induced pulmonary fibrosis, and this Cav-1 induction strongly correlates with improvements in the lung fibrosis score.^44^

Similar interactions were reported for Cav-1 and Hsp70. A knockdown of Hsp70 by siRNA strongly reduces mRNA and protein levels of Col1 and Col3, and also induces the MMP-2 expression in keloid-derived fibroblasts.^45^ At the same time, there is a negative correlation between protein Cav-1 and Hsp70 expressions in tubulointerstitial fibrosis.^46^ Hsp70 is also functionally involved in the cell surface localization of glycolytic enzyme alpha-enolase,^47^ which, in turn, is connected with Cav-1 and Annexin 2.^48^ A knockdown of these caveolae-associated proteins provides markedly decreased expression levels of alpha-enolase, which mediates an increased roughness of the plasma membrane and impairs the ability of cells to adhere to Col1 and Col4.^49^

These results demonstrate strong negative correlations between Cav-1 expression and functional HSPs, thereby explaining the remarkable overexpression of these proteins in fibro-proliferative disorders.

### Cav-1 involvement in the regulation of matrix metalloproteinases

Cav-1 is involved in remodeling of the extracellular matrix through interactions with different matrix metalloproteinases (MMPs).^50^ Suppression of Cav-1 was shown to activate expression of gelatinases MMP-2 and MMP-9, whereas the induction of Cav-1 causes suppression of these MMPs.^51^ This effect may be connected to the fact that MMP-2 and MT1-MMP are colocalized with Cav-1 on the cell surface.^52^

Whereas MMP-2 in mature HTS is strongly increased compared to normal tissue, the activity of MMP-9 in such scars is similar to normal skin.^53^ The highest activity of MMP-2 was found in keloids, followed by hypertrophic scars, normal skin, and atrophic scars.^54^ Myofibroblasts suppress the expression of the MMP-2 gene; moreover, expression of MMP-2 is inversely related to the level of α-SMA in these cells.^55^ This means that a potent increase in the activity of MMP-2 in HTS cannot simply be connected with the appearance of myofibroblasts in the wound. Interestingly, a long-lasting application of mechanical compression to HTS leading to clinical improvements in scar reduction correlates with an almost complete depletion of MMP-2 and a simultaneous increase of MMP-9 activity in HTS.^56^

Of note, MMP-2 is present but MMP-9 is absent in non-differentiated mesenchymal stem cells; on the other hand, MMP-2 expression is significantly reduced, whereas MMP-9 expression is highly increased in differentiating adipocytes.^57^ Thus, the observed expression of MMPs in HTS may be connected with the presence of non-differentiated mesenchymal stem cells in mature HTS and with the disappearance of these cells from HTS during the process of scar reduction. This means that dermis-WAT interactions may be an important feature in HTS formation.

Taken together, low expression of Cav-1 in the skin can promote local inflammation and induce fibro-proliferative conditions, leading to the production of HTS. This supports the recently proposed hypothesis that Cav-1 may be a potential therapeutic target for fibrosis.^58^

## Cav-1 expression and risk factors in HTS

Established and widely accepted risk factors for HTS include dark skin, female gender, young age, and burn site and severity.^2^ If we implicate a reduction of Cav-1 expression in enhanced HTS formation, these risk factors should be associated with a reduction of Cav-1 expression under conditions prone to HTS.

### Ethnic differences in Cav-1 expression

Indeed, cells obtained from a healthy African-American population demonstrate much lower Cav-1 content than the cells obtained from a Caucasians population, which correlates with higher rates of systemic sclerosis-related interstitial lung diseases in the African-American population.^59,60^ Moreover, enhanced expression of chemokine-receptors observed in lung monocytes isolated from African-Americans was driven by low Cav-1 expression.^61^ Recently, the same authors have reported that Cav-1 cooperates with the master adipogenic factor peroxisome proliferator-activated receptor gamma in maintaining a balance between fibrinogenic and adipogenic differentiation of precursors. This balance is biased towards a fibrogenic path both in subjects with systemic sclerosis and in healthy African-Americans.^62^ This lends further (though only correlational) support regarding involvement of the WAT in development of systemic sclerosis and dermal fibrosis.^23,63^

### Cav-1 expression in young and old cells

The expression of caveolins is significantly upregulated in chronological aging, and it was even suggested that Cav-1 reduction could serve as a potential anti-aging target.^64^ Substantial upregulation of Cav-1 in aging was observed in different tissues from 26-month-old rats as well as in human diploid fibroblasts.^65^ Lack of Cav-1 expression in lung fibroblasts dramatically inhibited premature senescence of these cells.^66^ Additionally, oxidative stress was shown to upregulate Cav-1 expression, which was connected with premature cellular senescence.^67^ This means that an increase of Cav-1 overexpression may be typical not only in chronological, but also in photo-induced aging. Indeed, UV-C irradiation (with fluence of 10 J/cm^2^) of mouse embryonic fibroblasts dramatically increased Cav-1 expression in these cells.^68^

### Sexual dimorphism in Cav-1 expression

Some authors reported that females compared to males demonstrate higher rates of HTS formation in the same body areas (odds ratios of 1.2–1.3),^69^ whereas others failed to attribute any sexual dimorphism for this process.^70^ These seemingly contradictory results can at least partly be explained by the fact that expression of Cav-1 is strongly dependent on the level of steroid hormones.^71^ Whereas expression of Cav-1 in abdominal WAT was shown to be slightly higher in control females than in males, application of steroid hormones reversed this relationship.^72^ Strong sexual dimorphism was reported also for Cav-1 expression in pulmonary hypertension^73^ and in osteoclastogenesis.^74^

Taken together, expression of Cav-1 is gender dependent, whereas its specific level is dependent on the body area and some additional parameters, such as the local levels of sexual hormones in the tissue.

### Burn site and severity

Burn site and its severity are known to significantly influence the probability of HTS development. Skin histology reveals a correlation between the presence of cone-like invaginations of the superficial WAT layer into the dermis and body areas that are typically susceptible to HTS formation,^75,76^ thereby explaining the anatomical site-dependent HTS occurrence as a function whether adipose tissue can undergo fibrosis or directly interact with the dermis.

Adipocytes can indeed locally interact with epithelial, endothelial and mesenchymal cells^23^ and at least some of these interactions involve Cav-1. For example, perivascular adipose tissue can induce enhanced Cav-1 expression in endothelial cells.^77^ Interfacial WAT is also directly involved in dermal fibrosis through induction of adipocyte-myofibroblast transition.^63,78^ In fact, the direct interactions between the dermis and dWAT leading to the substitution of adipose tissue by fibrotic structures is connected with a dysfunctional adiponectin pathway:^79^ in Cav-1^−/−^ mice, the secretion of adiponectin is reduced and the transmembrane signaling of adiponectin in endothelial cells is significantly blocked. Vice versa, the overexpression of adiponectin correlates with increased expression of Cav-1 in adipocytes.^80^ Moreover, in human adipocytes, adiponectin receptor 1 (AdipoR-1) interacts with Cav-1, producing an “AdipoR-1/Cav-1 signalsome”.^81^ In light of these observations, it is not surprising that the silencing of both adiponectin and Cav-1 leads to a severe inflammatory lung injury.^82^

To provide such interactions between the dermis and subcutis, skin injury must be deep enough to reach the dWAT layer. This can at least partly explain the dependence of the HTS formation on the depth of the skin burn.

**Fig. 1:**
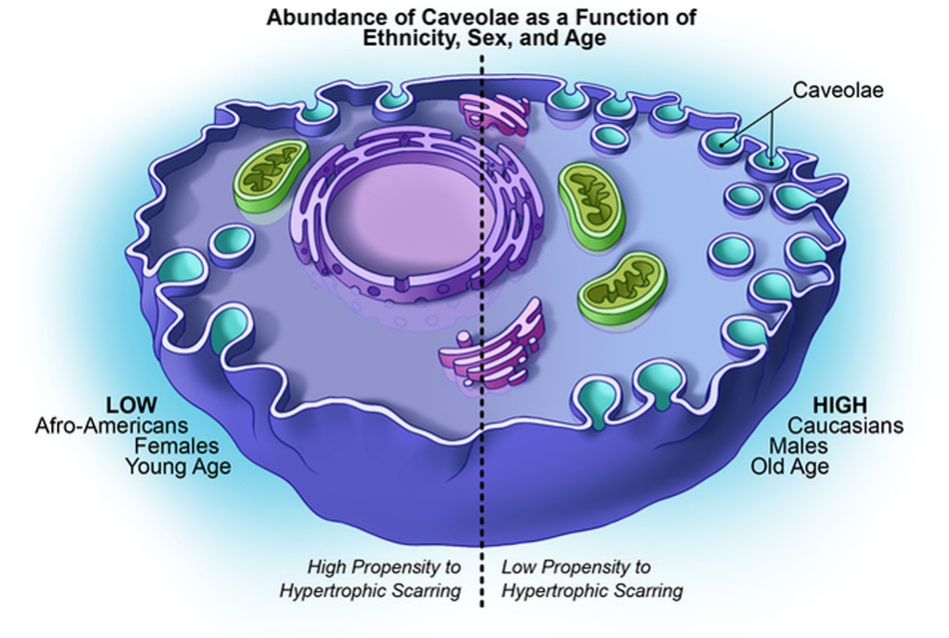
Possible role of Cav-1 in risk factors affecting HTS formation

Figure 1 summarizes the possible role of Cav-1 in risk factors affecting HTS formation.

## Cav-1 expression in therapeutic approaches to HTS treatment

Since deficient Cav-1 expression can both enhance the differentiation of myofibroblasts and induce the overexpression of extracellular proteins which can lead to the development of fibro-proliferative conditions in the skin, it seems obvious that endo- or exogenous induction of Cav-1 expression can improve HTS or even serve as preventive target to avoid its formation in the first place. Different treatment methods, including the application of static and dynamic mechanical forces, light-based technologies, various chemotherapeutics, corticosteroids, immunomodulators, anti-allergic drugs, and even hyaluronidase were reported to demonstrate some effectiveness in HTS treatment.^4,5^ Comparable effectiveness of such very distinct therapeutic options for the treatment of the same skin condition obviously suggests that these approaches must have similar targets which can be reached directly or indirectly. Further, the well-established therapeutic options in HTS have to be discussed from the viewpoint of their involvement in Cav-1 expression.

### Therapy options based on application of supra-physiological temperatures

Various light sources have been applied for the treatment of HTS, among them CO_2_, Er:YAG, Nd:YAG and PDL lasers, as well as intense pulsed light.^4,83,84^ Different authors have reported high levels of clinical improvements, including a significant height reduction in HTS. Since the wavelengths of these light sources broadly vary between 500 nm and 10,000 nm, the main impact of their application must be the heat transfer to the HTS. Indeed, thermography revealed that application of Nd:YAG laser causes a temperature rise in HTS up to 43–46 °C at a skin depth of about 0.5–1.0 mm.^83^

On the other hand, supra-physiological temperatures were shown to stimulate expression and re-localization of Cav-1. Mild hyperthermia can significantly increase expression of Cav-1 in different cells.^85,86^ Moreover, Cav-1 is internalized to the perinuclear region in NIH-3T3 cells at a temperature of 43 °C, but re-localizes to the plasma membrane after return to 37 °C.^87^

These results clearly demonstrate that application of supra-physiological temperatures enhances Cav-1 expression.

### Therapeutic options based on the application of drugs

For further discussion, it should be taken into account that the induced overexpression of Cav-1 can cause cell arrest of fibroblasts in the G0/G1 phase.^15,88^ Examination of fibroblasts obtained from normal skin as well as from 3-, 6-, 12- and 24-month old HTS reveals significant differences in the cell cycle distribution: whereas fibroblasts from normal skin and from old HTS (12- and 24-month) were predominantly in G0/G1 phase, fibroblasts from the 3- and 6-month old HTS were concentrated in the S and G2/M phases, respectively.^89^ Such a redistribution of cell cycles for fibroblasts during HTS maturation can significantly influence the cell cycle-specific effects of cytostatic drugs and thus modify clinical outcomes.

It is widely accepted that injections of corticosteroids can effectively improve HTS. Corticosteroids such as dexamethasone and triamcinolone acetonide were applied for the treatment of HTS and keloids. Their effects were explained by suppression of fibroblast proliferation through the TGF-β1 pathway.^90,91^ Since TGF-β1 is a negative regulator of Cav-1,^11,12^ we can expect that the application of these corticosteroids enhances Cav-1 expression. Indeed, dexamethasone in physiologically relevant concentrations induces Cav-1 expression in endothelial cells both at the mRNA and protein levels.^92^ Cav-1 is also essential for non-genomic actions of glucocorticoid receptors, since the dexamethasone effects were not observed in the Cav-1^−/−^ model.^93^

Bleomycin is a cytotoxic antibiotic which acts as a strong TGF-β suppressor. Cultured human dermal fibroblasts treated with bleomycin demonstrate reduced collagen synthesis even upon exogenous application of TGF-β1.^94^ Bleomycin is known to induce dermal and lung fibrosis accompanied with a dramatic reduction of Cav-1 in affected tissue.^95^ This pro-fibrotic effect of bleomycin is actually considered to be connected with the recruitment of new myofibroblasts from adipogenic progenitors^63,78^ and thus does not contradict its antifibrotic activity by superficial injections in HTS where such progenitors should be absent. On the other hand, bleomycin induces cell senescence and strongly increases expression of Cav-1 and -2 expression in epithelial lung cancer cells.^96^ Importantly, bleomycin induces significant changes in cell cycle distribution, shifting the Cav-1 positive cells from the G0/G1 into G2/M phase and producing irreversible cell cycle arrest. However, a knockdown of Cav-1 before the bleomycin treatment was able to prevent this effect.

Fluorouracil (5-FU) is a chemotherapeutic drug (inhibitor of thymidine synthase) which was reported to be effective in the therapy of keloids and HTS.^97^ In contrast to bleomycin, treatment with 5-FU demonstrated no effect on collagen synthesis in cultured human dermal fibroblasts.^94^ At the same time, 5-FU was able to inhibit fibroblast proliferation. 5-FU can also modulate Cav-1 expression: Cav-1 was strongly upregulated after breast cancer therapy with 5-FU both in vitro and in vivo.^98^ Very recently, it was reported that the downregulation of Cav-1 expression increases the cell sensitivity to 5-FU,^99^ which explains the high efficiency of this drug in HTS characterized by low Cav-1 levels.

Some other drugs which were successfully applied for the treatment of HTS demonstrate an interaction with TGF-β. Tranilast inhibits collagen production in cultured fibroblasts^100^ through suppression of TGF-β1 receptors.^101^ Imiquimod is an immune-modulator and agonist of toll-like receptor 7 (TLR7). TLR4 and TLR7 participate in fibrosis through the TLR-TGF-β-SMAD signal pathway, increasing the expression of TGF-β.^102^ Botulinum toxin A is also effective in HTS reduction^103^ and strongly suppresses the expression of TGF-β1 in fibroblasts derived from HTS.^104^ Taken together, suppression of the TGF-β1 pathway induced by these drugs generally leads to an increase in the expression of Cav-1^12^ which can significantly modify the structure of the HTS tissue.

Successful application of hyaluronidases (Hyal) for HTS treatment which was reported in ref. ^5^ can be connected with involvement of hyaluronan in linking the TGF-β receptors to caveolae^9^ and by the fact that endogenous hyaluronidases are known to counteract the TGF-β activity.^105,106^ Whereas TGF-β1 was shown to promote growth of mouse fibroblasts L929, this effect was almost completely suppressed in presence of Hyal-1 or -2.^105^ Stimulation of cells with TGF-β1 resulted in formation of the TGF-β1/Hyal-2 complex on the cell membrane followed by its internalization via endosomes.^106^ Correspondingly, it can be strongly assumed that exogenous hyaluronidase can also counteract TGF-β1, increasing its internalization and thus demonstrating anti-fibrotic activity.

Whereas no significant cancer risk was reported for the treatment methods discussed above, it should be noted that some primary tumors and metastasis exhibit high Cav-1 expression, which correlates with tumor progression and invasion. Theoretically, application of methods providing a strong local modulation of Cav-1 expression have the potential to be connected with some risk if pre-cancerous lesions are present in the treated area.

**Fig. 2:**
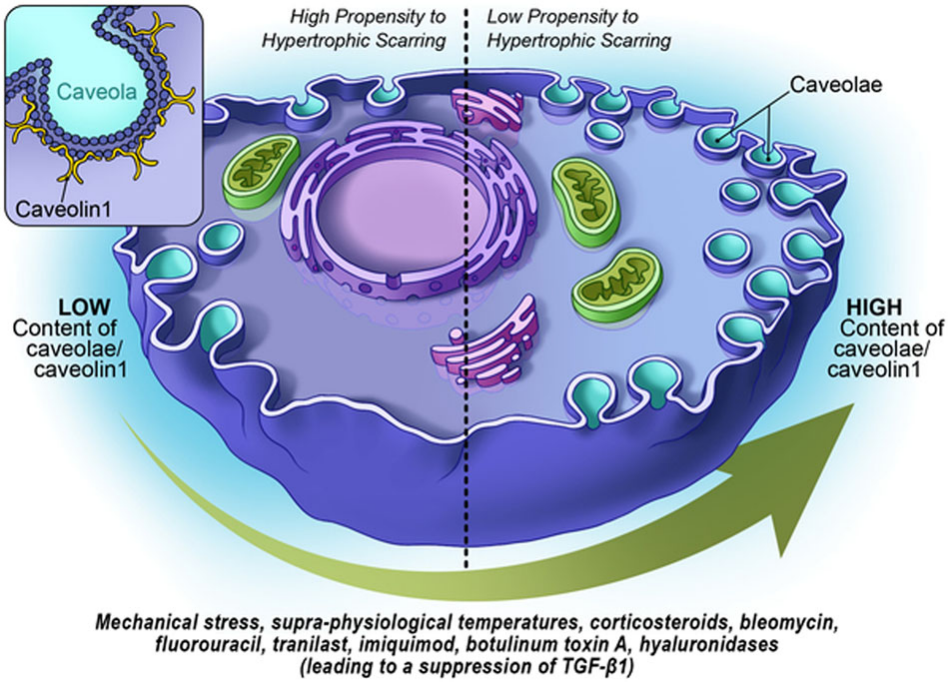
Cav-1 as a target in different therapeutic approaches to HTS treatment

Figure 2 summarizes the possible role of Cav-1 as a target in different therapeutic approaches.

## Cav-1 expression as a target in ultrasound treatment of HTS

Ultrasound is not considered as a modern therapy option in HTS because of mixed clinical outcomes and limited evidence for effectiveness. However, recent reports results demonstrate that this treatment option should be revisited.

Caveolae are linked to the actin cytoskeleton,^7^ and modification of the intracellular network strongly affects the surface density of these caveolar membrane structures. Application of a transient stretch to the cells can cause not only a stiffening of the cytoskeletal structures, but counterintuitively can also provide their softening and fluidization.^107,108^ Fluidization of the cytoskeleton is strongly dependent on the mechanical strain (relative deformation) amplitude, and the temporal behavior of such a system is also strain-rate dependent.^109^ Further, low-frequency (about 1 Hz) mechanical forces can effectively fluidize the cytoskeleton and modify the microdomain structure of the plasma membrane at strains of about 10%; at the same time, application of mechanical forces at frequencies of 1 MHz reduces the strain needed for fluidization of the cytoskeleton to about 10^−5^.^110^ Such behavior is typical for ultrasound waves where the amplitudes of the particles' displacement in the medium inversely depends on the frequency. Additionally, more recently, there was a report indicating that the application of higher ultrasound intensity and higher ultrasound frequencies induces higher levels of strain in cells, causing stronger fluidization of their cytoskeleton structure.^111^ It is however noteworthy that high-frequency ultrasound can induce expression of HSPs and that this expression is strongly ultrasound frequency-dependent.^112^

Recent findings make high-frequency ultrasound waves to an interesting modality for the modulation of the Cav-1 content in target tissues. Indeed, the application of ultrasound with a frequency of about 1 MHz and intensity of up to 2.5 W/cm^2^ significantly increases the expression of Cav-1 in HEp-2 cells in a time- and dose-dependent manner.^113^ The application of ultrasound with a frequency of 1.875 MHz and intensity of 0.25 W/cm^2^ also demonstrated the involvement of Cav-1 in the endothelial tissue response.^114^ Importantly, the modification of endothelial tissue observed in wildtype animals disappeared in Cav-1^−/−^ mice, which supports a direct involvement of Cav-1 in this process.

Additional effects of high-frequency ultrasound should be the induction of supra-physiological temperatures in HTS, which can also stimulate the expression and re-localization of Cav-1 in this tissue. Spatiotemporal distribution of temperatures and temperature gradients produced by ultrasound waves with frequencies of 3 MHz, 10 MHz, and 19 MHz in the skin and sWAT were recently investigated in ref. ^115^ Application of ultrasound of 3 MHz, 10 MHz, and 19 MHz with intensities of 1 W/cm^2^ for 10 s produced temperature rises in different depths of the skin of approximately 1–1.5 °C, 5–9 °C, and 8–16 °C, respectively. At the same time, ultrasound with a frequency of 19 MHz was able to produce high-temperature gradients of up to 14 °C/mm on the interface between skin and WAT. These thermo-mechanical properties produced by high frequency ultrasound build a theoretical foundation for applications of these waves for the treatment of fibro-proliferative diseases, especially of HTS.

## Conclusion and future directions

Reduced Cav-1 expression in the skin causes amplification of TGF-β signaling and enhanced differentiation of myofibroblasts leading to the overexpression of extracellular proteins and the development of fibro-proliferative conditions. This highlights Cav-1 prominently as an important factor in HTS pathogenesis. Reduced Cav-1 expression levels are a characteristic feature for the main known risk factors in HTS, such as dark skin, female gender, young age, burn site and its severity. Moreover, many therapeutic avenues for HTS are associated directly or indirectly with an increase in Cav-1 levels. This makes the endo- or exogenous induction of Cav-1 not only an important therapeutic goal for HTS treatment, but also highlights its potential as a preventive target to reduce or avoid HTS formation altogether. Altogether, we argue that more attention should be paid to different pharmacological and physical interventions that lead to an effective modulation of Cav-1 expression in HTS.
